# A lost cause

**DOI:** 10.1192/bjb.2018.1

**Published:** 2018-02

**Authors:** Anuradha Menon

**Affiliations:** Consultant Psychiatrist and Medical Psychotherapist, Leeds and York Partnership NHS Foundation Trust; email: anuradha.menon@nhs.net

On reading Dr Moorey's earnest response to Dr Gipps’ views, I was struck by his description of the ‘depressive mode’.^1^ This marvel of development, 100 years on from Freud's classic paper,^2^ is – in Dr Moorey's view – a ‘complex neural network, including multiple relevant brain regions that are activated or deactivated during depression’. This, he argues, is the target of therapeutic practice in cognitive–behavioural therapy (CBT), where unconscious schemas are automatic, not repressed. It seems to the reader that in this dehumanised framework, grief and loss are merely ‘problems’ that face humankind, which need to be put on the CBT table to be sorted out openly between therapist and patient. The tools? Good old-fashioned common sense, an indefatigably optimistic therapist and well-positioned intelligence. As for the measures: specially designed scales that measure the very structure which they helped create.

I am writing this piece to explore how both Dr Moorey and Dr Gipps warily circle around a point which is never highlighted in its own right.

Freud's theory of melancholia^2^ posits an unconscious basis for the depressed patient's dilemma. This theory holds true: the proof is in the analytic setting, and all contemporary psychoanalytic approaches which describe the transference find their roots in this classic paper.^3^ Freud writes about an identification of the ego with the abandoned object, saying ‘thus the shadow of the object fell upon the ego’. This is still pre-object relations. American psychoanalysis, which was evolving at the time, is well known to have been profoundly influenced by Freud.

In the 1950s, psychoanalysts around the world were working on extending early theories, and the Americans actively participated in this worldwide development. So, with respect, Dr Moorey's point about Beck's ‘perfectly acceptable masochism hypothesis’ is a bit like Lamarck chasing after amputated tails while Darwin was thinking about evolution!

The crux of the matter is that Beck moved into what is essentially the conscious realm when he developed his theory. Today, anyone who manages patients with depression will know that the latter is the easier and, dare we say, cheaper option. It's all backed up by robust evidence, and supported by what is essentially an Orwellian^4^ environment. I hardly need wonder why patients are rarely asked what they prefer: an analyst who is interested in undiscovered aspects of their loss and is willing to explore themselves in the process, or one bent on pinning down the patient's experience in prosaic terms.

This is why I take exception to the statement in Dr Moorey's final paragraph, which is unreferenced and states that CBT ‘has given psychoanalysts methodologies they now use to evaluate their own theories’. This is outrageous, as no self-respecting psychoanalyst would turn to a two-dimensional construct to tell them whether they are tuned into their patient's inner world. I think this is another example of the kind of empiricism that undermines a patient's personal struggle with loss, ignores the depth in a poorly understood psychoanalytic theory and exposes a flawed theoretical argument. Why would psychoanalysis have any real use for a methodology that doesn't even address its basic theoretical stance? Dr Moorey's thesis is, unfortunately, a lost cause. So, to quote from the excellent choice of title for this debate:

‘I give you the mausoleum of all hope and desire […] I give it to you not that you may remember time, but that you might forget it now and then for a moment and not spend all of your breath trying to conquer it. Because no battle is ever won he said. They are not even fought. The field only reveals to man his own folly and despair, and victory is an illusion of philosophers and fools’. (William Faulkner, *The Sound and the Fury*^5^)
